# MicroRNAs and exosomes: promising new biomarkers in acute myeloid leukemias?

**DOI:** 10.31744/einstein_journal/2022RB5954

**Published:** 2022-02-23

**Authors:** Helena Varela de Araújo, Luis Henrique Toshihiro Sakamoto, Nydia Strachman Bacal, Sidnei Epelman, Juliana Monte Real

**Affiliations:** 1 Centro de Hematologia de São Paulo São Paulo SP Brazil Centro de Hematologia de São Paulo, São Paulo, SP, Brazil.; 2 Núcleo Avançado de Pesquisa em Oncologia Hospital Santa Marcelina Faculdade Santa Marcelina São Paulo SP Brazil Núcleo Avançado de Pesquisa em Oncologia, Hospital Santa Marcelina, Faculdade Santa Marcelina, São Paulo, SP, Brazil.

**Keywords:** Leukemia, myeloid, acute, Exosomes, MicroRNAs, Immunomodulation, Biomarkers

## Abstract

Despite advances in understanding of carcinogenesis and of treatment of acute myeloid leukemia, this neoplasm still has a lethality of at least 30%. The search for biomarkers that can predict the response to treatment in the early stages of the disease is still necessary. In recent years, a new form of cellular communication between tumor and non-neoplastic cells has been discovered: the exchange of information through extracellular vesicles. These are small vesicles released by membrane-coated cells that carry proteins, lipids, messenger RNAs, microRNA and DNA, which can be internalized and promote biological changes in target cells. Exosomes are qualified as a type of extracellular vesicle and, in tumors, carry immunoinhibitory signals that promote the escape of immune control. Recent studies have showed their involvement in communication with the cells of the tumor microenvironment and with chemoresistance in several tumors. To date, there is no information about immunoregulatory microRNAs transported by exosomes and their correlation with clinical evolution during chemotherapy for acute myeloid leukemia. Knowledge about immunomodulatory microRNAs obtained by leukemic cells and transported by exosomes can direct us towards the design of new diagnostic and treatment tools in this type of leukemia.

## INTRODUCTION

The overall survival rates for adult and childhood acute myeloid leukemia (AML), in addition to being suboptimal, have been stagnant for more than 20 years. This demonstrates the efficacy of chemotherapeutic regimens appears to have reached its upper limit. The need for the search for new therapeutic weapons against the disease is urgent. The way in which the tumor cell acts on the immune system and its ability to produce exosomes that influence the tumor niche has drawn attention of the scientific community. Thus, the transfer of non-coding RNAs by exosomes and the modulation of gene expression in target cells present new horizons to be explored.

## ACUTE MYELOID LEUKEMIA

Acute myeloid leukemia is a hematologic malignancy of complex pathogenesis, characterized by abnormal proliferation and differentiation of clonal populations of myeloid stem cells in the bone marrow.^(1)^

The worldwide incidence of AML is 2.5 to three new cases per 100,000 individuals per year, and although it can occur at any age, it is most commonly diagnosed in individuals aged over 65 years.^(1)^ In children under 15 years of age, it accounts for only 15% to 20% of acute leukemia cases.^(1)^

Some genetic and environmental risk factors seem to be associated with a higher frequency of AML. Among the constitutional genetic syndromes that may be correlated with AML, Down syndrome, Fanconi anemia, Blackfan-Diamond anemia, Shwachman-Diamond syndrome, and congenital neutropenia (Kostmann syndrome) stand out. Among the environmental factors, exposure to ionizing radiation, alkylating agents, and topoisomerase-II inhibitors are of epidemiological importance.^(1)^

The treatment protocol of the Berlin-Frankfurt-Münster (BFM) Group is the most widely used for treatment of AML in Brazil. The treatment is based on induction chemotherapy, with the infusion of cytarabine (ARA-C), idarubicin, and etoposide (VP-16), in addition to the administration of intrathecal cytarabine.^(2)^Despite the high remission rate, 90% of patients relapse if they do not continue the protocol with consolidation and intensification. Consolidation is performed in patients classified as low risk on the 15^th^ day of induction, using prednisone, thioguanine (Lanvis™), vincristine, doxorubicin (Adriamycin), cytarabine, intrathecal cytarabine, cyclophosphamide, and granulocyte colony-stimulating factor (G-CSF).^(2)^The other patients start consolidation after high-dose cytarabine and etoposide intensification, while low-risk patients perform intensification after consolidation.^(2)^

A considerable part of the increase in survival rates of these patients, however, is due to the improvement in supportive treatment for the complications of chemotherapy. Allogeneic hematopoietic stem cell transplantation in the first remission is also an option, especially for those who are poor responders to induction therapy or who have poor prognostic cytogenetic and molecular characteristics.^(2)^

Despite the advances made in the last 40 years, the survival rates for AML are less than desirable, remaining around 70% at 5 years, even for good responders to induction therapy.^(2)^ New therapies are already being used for gene variants of AML, such as FLT3 (midostaurin and gilterinib), BCL2 (venetoclax), and IDH1/IDH2 (ivosidenib and enasidenib) inhibitors.^(3)^ However, such alternative therapies are not yet widely used, either because of their cost or because they require approval for use in Brazil.

## WHAT IS KNOWN ABOUT EXTRACELLULAR VESICLES, EXOSOMES, AND MICRORNAS IN ACUTE MYELOID LEUKEMIAS?

Extracellular vesicles are secreted by almost all cell types and have the function of performing intercellular communication, which can occur through ligand-receptor interaction, by fusion, and/or internalization.^(4,5)^ As mediators of intercellular communication, the extracellular vesicles can transmit proteins and lipids, besides different types of RNAs and DNA.^(4)^

Extracellular vesicles are classified according to their size into exosomes (30nm-150nm), microvesicles (0.1μm-2μm) and apoptotic bodies (0.1μm-5μm).^(4,6)^ Recently, exosomes were subclassified into Exo-L (large exosome vesicles) and Exo-S (small exosome vesicles), with 90nm-150nm and 60nm-80nm, respectively. In addition, the term “exomere” was created for classification of non-membranous nanoparticles with approximately 35nm.^(6)^

Besides being differentiated by size, extracellular vesicles have different densities and origins. While microvesicles and apoptotic bodies are formed from the direct evagination of the plasma membrane, exosomes are formed from a more complex process, which involves the incorporation of protein or molecular content by invagination into cytoplasmic multivesicular endosomes.^(4)^ Subsequently, these endosomes are fused to the plasma membrane, releasing the exosomes into the extracellular environment. As a consequence of their endosomal origin, exosomes contain proteins involved in membrane transport and fusion (*e.g*., Rab GTPases and annexin), as well as in the biogenesis of multivesicular bodies (*e.g.*, TSG101), and integrins and tetraspanins (*e.g.*, CD9, CD63, CD81, and CD82).^(7)^

Various physiological and pathological stimuli can increase the production of extracellular vesicles, which can be detected in body fluids. This feature has opened the horizons for investment in research aimed to use these changes as possible biomarkers in various diseases.^(7,8)^

With respect to exosomes, tumor-derived exosomes are known to carry immunoinhibitory signals that promote escape from immune control. Evidence shows that tumor cell-derived exosomes affect proliferation, apoptosis, and cytokine production, and are able to reprogram cells of innate and adaptive immunity. It has been described, for example, that exosomes containing miRNA-21-3p, miRNA-185d-5p, and miRNA-1246 have the potential to reprogram macrophages into tumor support agents. Furthermore, exosomes expressing transforming growth factor β1 (TGF-β1) and binding to NKG2D may downregulate NKG2D expression, reducing natural killer (NK)-induced cytotoxicity.^(6)^Moreover, exosomes are involved in communication with cells in the tumor microenvironment and chemoresistance in several solid neoplasms.^(7,8)^

Exosomes can carry inside them microRNAs (miRNAs), small endogenous non-coding RNAs (19 to 25 nucleotides), originally identified as regulators of larval development in *Caenorhabditis elegans*.^(9)^ Later, they were also associated with several types of cancer and with various cellular processes, such as DNA methylation, cell growth, differentiation, and apoptosis.^(10)^ Abnormal levels of these miRNAs may play a role in carcinogenesis, since certain overexpressed miRNAs can lead to silencing of tumor suppressors.^(10)^The imbalance of these miRNAs has been described in several types of cancers and in hematological neoplasms. Additionally, miRNAs can induce molecular alterations in target cells, affecting the regulation of specific genes, making it pathologically possible to alter processes, such as differentiation, proliferation, and apoptosis.^(11)^

Recent studies suggest that extracellular vesicles carry an important amount of non-coding RNA that is relevant to the pathogenesis of leukemias. Some miRNAs have already been described as involved in the pathogenesis of AMLs, as reviewed by Trino et al.,^(12)^ and Li et al,.^(13)^ as shown on table 1.

**Table 1:** MicroRNAs as prognostic biomarkers in acute myeloid leukemia

miRNA	Expression	Prognostic impact	Biological sample
miRNA-9	Hyperexpression	Unfavorable OS and RFS	BMMCs
miRNA-24	Hyperexpression	Shorter OS	BMMCs/PBMCs
miRNA-26a, miRNA-29b, miRNA-146a	Hyperexpression	Shorter OS	BMMCs
miRNA-29a	Hypoexpression	Shorter OS and RFS	BMMCs
miRNA-29b	Hypoexpression	Worse OS	BMMCs/PBMCs
miRNA-34a	Hypoexpression	Worse OS and RFS	
miRNA-96	Hypoexpression	Shorter OS and RFS	BMMCs/PBMCs
miRNA-99a	Hyperexpression	Worse OS and EFS	BMMCs
miRNA-124-1	Hypoexpression	Longer SO and RFS	BMMCs
miRNA-126	Hyperexpression	Worse prognosis	
miRNA-135a, miRNA-409-3p	Hypoexpression	Largest cumulative incidence of relapse	BMMCs/PBMCs
miRNA-133	Hyperexpression	Increased sensitivity to doxorubicin	
miRNA-150, miRNA-155	Hyperexpression	-	
miRNA-181a, miRNA-181b	Hyperexpression	Decreased risk of an event (*e.g.*, lack of complete remission, relapse, or death)	BMMCs
miRNA-181a	Hyperexpression	Favorable prognosis	BMMCs
miRNA-181a, miRNA-181b, miRNA-181d	Hyperexpression	Longer OS	Not specified
miRNA-181a	Hyperexpression	Higher rate of CR and longer OS	BMMCs
miRNA-181b	Hyperexpression	Lower CR, and shorter RFS and OS	BMMCs
miRNA-181b	Hyperexpression	Lower rates of CR, shorter RFS and OS	BMMCs
miRNA-188-5p	Hypoexpression	Longer OS and EFS	Not specified
miRNA-191, miRNA-199a	Hyperexpression	Worse prognosis (worse OS and EFS)	BMMCs
miRNA-193a	Hyperexpression	Repression of the expression of c-kit	
miRNA-196b	Hyperexpression	Shorter OS	Leukemic blasts
miRNA-196b, miRNA-644	Hyperexpression	Shorter OS	BMMCs/PBMCs
miRNA-204	Hypoexpression	Worse prognosis	
miRNA-212	Hyperexpression	Longer OS, higher CR rate, and better EFS and RFS	BMMCs/PBMCs
miRNA-216b	Hyperexpression	Shorter OS	
miRNA-328	Hypoexpression	Shorter OS and RFS	Plasma
miRNA-331	Hyperexpression	Worst response to therapy and shorter OS	BMMCs
miRNA-338	Hyperexpression	Worse OS and EFS	
miRNA-375	Hyperexpression	Shorter RFS and OS	BMMCs
miRNA-378	Hyperexpression	Shorter RFS	BMMCs
miRNA-3151	Hyperexpression	Shorter DFS and OS	PBMCs
let-7a-3	Hyperexpression	Shorter OS and RFS	BMMCs
let-7a-2-3p	Hyperexpression	Longer OS and EFS	
let-7d	Hypoexpression		
Circulating miRNA-10a-5p	Hyperexpression	Shorter OS	Serum
Circulating miRNA-181d-5p	Hyperexpression	Worse OS	Serum
Circulating miRNA-183	Hyperexpression	Shorter OS and RFS	Serum
Circulating miRNA-210	Hyperexpression	Shorter OS and RFS	Serum
Circulating miRNA-335	Hyperexpression	Shorter OS and RFS	Serum
Circulating miRNA-370	Hypoexpression	Shorter OS and RFS	Serum

Source: Trino S, Lamorte D, Caivano A, Laurenzana I, Tagliaferri D, Falco G, et al. MicroRNAs as new biomarkers for diagnosis and prognosis, and as potential therapeutic targets in acute myeloid leukemia. Int J Mol Sci. 2018;19(2):460. Review^(12)^ and Li M, Cui X, Guan H. MicroRNAs: pivotal regulators in acute myeloid leukemia. Ann Hematol. 2020;99(3):399-412. Review.^(13)^

miRNA: microRNA; OS: overall survival; RFS: relapse-free survival; BMMC: bone marrow mononuclear cell; PBMC: peripheral blood mononuclear cell; EFS: event-free survival; DFS: disease-free survival; CR: complete remission.

Exosomes originating from leukemic cells in AML patients have a distinct molecular profile when compared to healthy individuals.^(14)^ In addition to conventional markers for exosomes such as tetrapanins, AML exosomes contain TGF-β1, MICA/MICB, FasL, and markers of myeloid blast cells (CD33, CD34, and CD117).^(14)^*Ex vivo,* these exosomes decrease NK cell cytotoxicity by decreasing expression of NKG2D.^(14)^ In addition, TGF-β1 is partially responsible for NK cell dysfunction in AML. Thus, it can be said that exosomes derived from leukemic cells in AML have the potential to modulate the immune system and may influence disease progression as well as response to therapy.^(14)^A summary of functional studies demonstrating the relation between altered exosome production and AML is shown on table 2.^(15-19)^

**Table 2:** Review on functional studies of exosomes in acute myeloid leukemia

References	Source of exosome	Receptor cell	Load transported	Biological consequence
				Effects on stromal cells
Huan et al.,^(15)^	Human AML cell line (Molm-14)	Murine B-cell precursor cell line (Ba/F3)	miR-150	Reduced migration and decreased expression of the surface protein CXCR4 (target of miR-150)
Huan et al.,^(16)^	Human leukemic cells in NSG* mouse xenografts	Hematopoietic progenitor stem cells	Not identified	Decreased clonogenicity, loss of CXCR4 and c-Kit expression, repression of hematopoiesis-related transcription factors
Kumar et al.,^(17)^	Human AML cell lines	Mesenchymal BM cells	Not identified	Increased DKK1 (osteogenesis suppressor) preconditioning with AML-derived exosomes favored tumor growth in mice
				Sensitivity to treatment
Viola et al.,^(18)^	BMSC of AML and healthy patients	Human AML cell line (Molm-14)	miR-155, miR-375, TGF-β1, IL-8	AML-BMSC and normal-BMSC exosomes promote chemoresistance of tumor cells to cytarabine; AML-BMSC exosomes protect against AC220 treatment
				Immune response
Hong et al.,^(19)^	Plasma from refractory/relapsed AML patients	NK-92 cells	CXCL4, CXCL7, CCL5, TGF-β	Inhibited NK-92 cell functions *ex vivo* and decreased its antileukemic activity

* NSG: immunodeficient mice (Jackson Laboratory^®^).

AML: acute myeloid leukemia; BM: bone marrow; BMSC: bone marrow stromal cells; TGF-β: transforming growth factor beta; IL: interleukin; NK: natural killer.

The first study to show that AML cell lines release exosomes was published in 2013.^(15)^ Exosomes appear to be involved in the deregulation of hematopoiesis during the conversion from a homeostatic microenvironment to a leukemic niche. In the *in vivo* model,^(16)^exosomes of AML cell lines have been observed to migrate to the bone marrow, decreasing the colony-forming capacity of hematopoietic progenitor stem cells, and the expression of several transcription factors of genes involved in hematopoiesis.^(16)^ The influence of extracellular vesicles on the tumor niche was also observed by Kumar et al.,^(17)^ bone marrow preconditioning with exosomes from AML cells accelerated tumor engraftment and growth, promoted increased expression of DKK1 in bone marrow (a negative regulator of osteoblastic development), and caused reduced trabecular bone volume.^(17)^ In patients with AML, plasma levels of the bone formation indicator gene (OCN) are decreased.^(17)^ Interestingly, the number of circulating exosomes in plasma is significantly increased in patients with AML when compared to healthy individuals.^(17)^ In view of these findings, the authors emphasize the potential need to treat the induced changes in the bone marrow stroma as well, and not only to eradicate leukemic cells. Viola et al.,^(18)^ corroborated these observations and showed that monocyte-derived exosomes from patient stroma protect the AML cell line against chemotherapy treatment with cytarabine or FLT3 inhibitor.^(18)^

Among the investigations that evaluated the content of extracellular vesicles in AML, miRNA in exosomes circulating in plasma^(20)^ and released by bone marrow monocytes,^(21)^ and apoptotic proteins found in exosomes derived from bone marrow blasts have been described.^(22)^ In patients with AML refractory to treatment with adoptive cell therapy using NK-92 cells, exosomes isolated from plasma have been shown to reduce the cytolytic activity of NK-92 cells and to decrease their migration capacity.^(19)^ Exosomes have also been found to carry TGF-β and PD-L1 (programmed cell death ligand 1), an important T cell inhibitor.^(19)^

In addition, AML blasts can secrete extracellular vesicles that express myeloid cell line-specific markers distinct from those present in normal cells. The expression of CD13, CD34, CD117, and CD33 on extracellular vesicles isolated from plasma of AML patients may indicate the presence of active leukemic cells.^(23)^ Thus, in clinical practice, flow cytometry assays may incorporate the search for extracellular vesicles as biomarkers of disease activity.

## COMMENT

Although some studies have already evaluated some of the content of extracellular vesicles at specific times in acute myeloid leukemia, no research has shown whether there is modification of this content over the course of treatment. In addition, some immunoregulatory microRNAs have not yet been evaluated and, perhaps, they may have relevance in the pathogenesis of acute myeloid leukemia. Likewise, very little is known about the dynamics of extracellular vesicles in childhood leukemias.

The importance of circulating exosomes and cellular communication by microRNA in acute leukemias remains poorly elucidated, but recent studies^(24)^ indicate the possibility that in the future, extracellular vesicles will be used as a substrate in laboratory assays for patient follow-up, prognosis determination, and therapeutic response.
